# Orthogonal Experimental Study on Concrete Properties of Machine-Made Tuff Sand

**DOI:** 10.3390/ma15103516

**Published:** 2022-05-13

**Authors:** Dunwen Liu, Wanmao Zhang, Yu Tang, Yinghua Jian, Yongchao Lai

**Affiliations:** School of Resources and Safety Engineering, Central South University, Changsha 410083, China; dunwen@csu.edu.cn (D.L.); 205512136@csu.edu.cn (W.Z.); jyh__0412@csu.edu.cn (Y.J.); 215512141@csu.edu.cn (Y.L.)

**Keywords:** machine-made sand, concrete, orthogonal experimental design, grey correlation analysis, compressive strength

## Abstract

Machine-made sand instead of natural sand has become an inevitable choice for the sustainable development of the concrete industry. Orthogonal experiment and grey correlation analysis were used to investigate the performance of machine-made tuff sand concrete. The optimal concrete mix ratio of machine-made sand was obtained by orthogonal test and its working performance was verified. Grey correlation analysis was applied to compare the factors affecting the mechanical properties of the machine-made sand concrete. The test results show that the sand rate has the greatest degree of influence on slump and slump expansion. The mineral admixture has the greatest effect on the 7-day compressive strength of the concrete. Additionally, the water–cement ratio has the greatest influence on the 28-day compressive strength. The mechanical and working properties of the machine-made sand concrete reach the optimum condition when the mineral admixture is 20%, the sand rate is 46%, the stone powder content is 10% and the water–cement ratio is 0.30. Comparing different fine aggregate concretes of similar quality, we conclude that the mechanical and working properties of tuff sand concrete and limestone sand concrete and river sand concrete are similar. The compressive strengths of the mechanism concrete show the greatest correlation with roughness and the least correlation with stone powder content. The stone powder content has almost no effect on the compressive strength of concrete when the stone powder content does not exceed a certain range. The results of the study point out the direction for the quality control of concrete with machine-made sand.

## 1. Introduction

Concrete is the most widely used material for building structures today. As China invests more in infrastructure construction, concrete consumption is still on the rise. Fine aggregates account for about one-third of the raw materials for concrete. Fine aggregates can be divided into natural sand and artificial sand [1]. River sand is the most widely utilized natural sand. In recent years, the production of natural sand is no longer able to meet the demand for sand in the construction industry. There is an urgent need to find other materials to replace natural sand [2]. In the middle of the last century, the United States and other developed countries began to apply machine-made sand in actual engineering. China started to use machine-made sand in an orderly and standardized way in the late 1970s. In China, the most-used artificial sand is machine-made sand. Compared with ordinary river sand, it has many advantages such as controlled raw material, stable performance, and low cost. It is suitable for promotion and application in the construction industry [3,4]. The raw materials of machine-made sand mainly include mine waste stone, tunnel excavation waste stone, mountain stone and pebbles. It is obtained from these raw materials by crushing, screening and dedusting.

There are many methods for designing the proportioning of machine-made sand concrete, and different proportioning methods have their own focus [5,6]. They are designed to meet specific performance requirements [7,8]. On the other hand, it is due to practical conditions such as raw materials and environmental conditions in the area [9]. The excellent quality of fine aggregates largely determines the concrete performance. Shen et al. [10] invented a method for the design of the mix ratio based on gradation optimization and volumetric analysis. Additionally, the ultra-high strength concrete of C120 grade was successfully prepared with sandstone machine-made sand. Ji et al. [11] found an extreme point where the compressive strength of concrete as the content of fines in the cementitious material increases from 0% to 30%. Li et al. [12] proposed that the stone powder in the machine-made sand has the effect of filling the pores of concrete and improving the gradation of aggregate particles. Menadi, Topcu and Eren et al. [13,14,15] proposed that the compressive and flexural strengths of the concrete with mechanism sand reached the maximum at 10% of stone powder. Above or below 10%, the machined sand concrete does not perform its best mechanical properties. Munoz et al. [16] compared the effect of various clay powders on strength. The effect of sodium montmorillonite and calcium montmorillonite on strength was significantly greater than that of kaolinite and illite. Chen et al. [17] formulated machanism sand concrete by replacing cement slurry with limestone rock powder in proportion to its volume. It was found that the incorporation of stone powder could significantly increase the strength of concrete at a fixed volume of slurry with different water–cement ratios. Felekoglu [18] compared washed river sand, washed machine-made limestone sand, machine-made limestone sand mixed with clay powder and machine-made limestone sand mixed with clay. Chouhan et al. [19] studied the effect of limestone waste on mechanical properties and durability of concrete. Partial or total replacement of concrete fine aggregates with waste materials resulted in increase in compressive and tensile strength of the colluvial sand specimens with the rise in waste material admixture. The durability and bonding properties of the specimens were also improved. Bravo et al. [20] carried out an in-depth analysis of the properties of natural and recycled aggregates. Compressive strength, splitting tensile strength, modulus of elasticity and abrasion resistance tests were also carried out. Wang et al. [21,22] studied the effect of different methylene blue (MB) values on the performance of low and high strength machine-made sand concrete. It was found that under fixed cement dosage, water–cement ratio, and water-reducing agent admixture, the compressive strength at each age increased and then decreased with increasing MB value when MB value > 1.8 g/kg. The impermeability performance showed a different pattern.

Today, the complexity of civil engineering has prompted the industry to develop a variety of new materials. In line with the concept of sustainability, the construction industry is trying to replace ordinary silicate cement concrete using geopolymer concrete [23,24,25]. Similarly, the formulation of high-performance concrete from machine-made sand is one of the concrete material sustainability studies [26,27]. Due to the wide distribution of limestone machine-made sand, a large number of studies focus on machine-made limestone sand [28,29,30,31], and less research on machine-made sand of other lithologies. River sand resources are scarce in the region of eastern China, which restricts the development of local engineering construction. However, tuff is abundant and the raw materials for sand making are widely available, which also includes tunnel rejects. The use of tuffaceous sand concrete in engineering practice is relatively small [32]. Additionally, it’s working state and mechanical properties in engineering structural components are worth exploring in depth [33,34]. In addition, due to the large quality fluctuations in the production process of machine-made sand, there are fewer studies on the effect of machine-made sand quality on the performance of concrete [35]. Therefore, further research is needed on the effect of quality variation of machine-made sand of different lithologies on performance.

Therefore, in this study, the research on machine-made tuff sand concrete is carried out through the following aspects. Firstly, the factors influencing the high-performance machine-made sand concrete mix ratio are analyzed. These factors to be optimized are used as influencing factors for orthogonal tests. The test results are analyzed to find the optimum level of concrete performance under each influencing factor. The optimal machine-made sand concrete ratio is obtained, and the performance of concrete under the optimal ratio is verified. We used the grey correlation analysis to calculate the correlation between fine aggregate stone powder content, clay lump content, roughness and compressive strength. The main factors affecting the mechanical properties of the machine-made sand concrete were analyzed. Then, we compare the correlation matrices of tuff and limestone machine-made sand. The magnitude and laws of different factors on the concrete properties are analyzed to show the direction for the quality control of the machine-made sand concrete.

## 2. Materials and Methods

### 2.1. Materials

**Figure 1:**
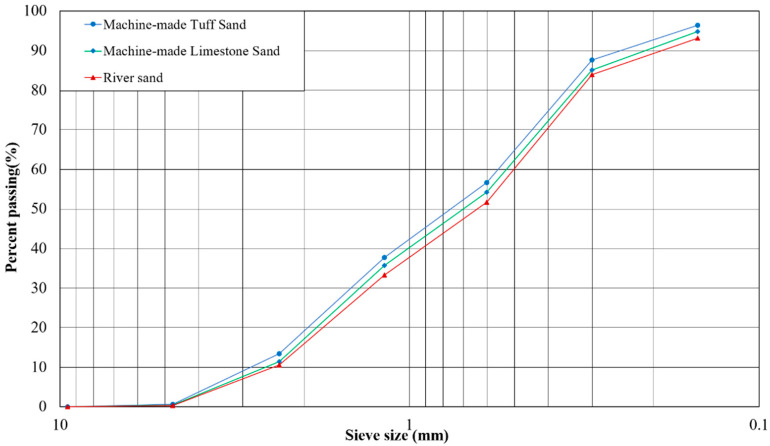
Fine aggregate grading curve.

In this study, P.O 42.5 grade ordinary silicate cement was used for the cement. Its 28-day flexural strength was 7.5 MPa and compressive strength was 46.8 MPa, specific surface area was 348 m^2^/kg. Fly ash used Grade II fly ash with a fineness of 45 μm sieve margin of 15.2%, a burn-off of 1.78%, and a water demand ratio of 101%. Slag powder using S95 grade slag powder, specific surface area 411 m^2^/kg, 7-day activity index 83%, water content 0.85%, flow rate ratio of 98%. Water reducing agent is selected from a high efficiency water reducing agent. Polycarboxylic acid water reducing agent has the characteristics of low dosing and high water-reduction rate. The water reduction rate is 25%. In this study, the dosing range is 1.2%~1.8% of the cementitious material. The experimental mixing water is tap water, which meets the requirements of Chinese national standards. The fine aggregates are selected from machine-made tuff sand (number NH), machine-made limestone sand (number SH) and river sand (number HS). The physical index of fine aggregate is shown in Table 1. The coarse aggregate is tuffaceous gravel with nominal particle size of 5~20 mm, continuous gradation, apparent density of 2.63 g/cm^3^ and bulk density of 1.52 g/cm^3^. The gradation curve of fine aggregate is shown in Figure 1.

### 2.2. Orthogonal Experimental Design

Orthogonal experimental design is a quantitative design analysis method for studying multiple factors and levels. This experiment method is commonly used to optimize mortar and concrete properties [36,37]. There are many factors affecting the performance of the machine-made sand concrete, and the number of experiments is large if all the factors are experimented. The factors that have a greater influence on the mix ratio are derived from the analysis and evaluation. The proportion of mineral admixture, stone powder content, sand rate, and water–cement ratio are selected as orthogonal test design factors, which are denoted by the symbols A, B, C, and D. The mineral admixture type is also a factor to be optimized. The only mineral admixture in the calculated ratio is fly ash. Single mineral admixture is not conducive to the development of concrete properties, and the subsequent tests used fly ash and slag powder compounding [38,39]. Machine-made sand was first removed by dry sieving and then mixed with tuff stone powder to adjust the stone powder content of fine aggregates.

We used the full calculation method to perform preliminary tests on the properties of the machine-made sand concrete. The mix ratio was optimized by considering the level of factors and the number of tests. Four levels were set for each factor, and a 4-factor, 4-level orthogonal test table was selected for the experiments. The parameter values under each level are shown in Table 2, the experimental scheme is shown in Table 3, and the amounts of raw materials under each test scheme are shown in Table 4.

### 2.3. Comparison of Fine Aggregate Performance Test

Three different types of fine aggregates, machine-made tuff sand, machine-made limestone sand and river sand (number NH, SH, HS), were selected and tested in different batches of the same fine aggregate. Additionally, the fineness modulus, bulk density, apparent density, clay lump content and roughness of each batch were measured. Four batches of machine-made tuff sand (number NH1, NH2, NH3, NH4), four batches of machine-made limestone sand (number SH1, SH2, SH3, SH4), and two batches of river sand (number HS1, HS2) were sieved to obtain two types of stone powder to adjust the stone powder content of the sand. The stone powder content of each batch of the two types of sand was set to 3.0%, 6.5%, 10.0% and 13.0%. The clay lump content of the batches of the two types of machine-made sand fluctuated widely from 0.2% to 0.9%. The clay lump content of river sand was higher at 0.7% and 0.9%. The performance indicators of each batch of fine aggregate meet the corresponding national standards, and the main performance indicators are shown in Table 5.

The degree of influence of index properties such as stone powder content, clay lump content and roughness of the machine-made sand on the mechanical properties of concrete is different. The compressive strengths of concrete with different fine aggregates were tested and obtained through comparative studies of concrete with different batches of tuff and machine-made limestone sand and river sand. The correlation between the stone powder content, clay lump content, roughness of the machine-made sand and compressive strength of the concrete was also calculated by using the grey correlation analysis. The correlation matrix was analyzed to derive the magnitude and influence law of each factor on the degree of performance.

### 2.4. Test Methods

The concrete strength grade in this study is C50, which is mainly applied to the T-beam in the bridge structure. The T-beam is the main component of the main structure of the bridge and has a design service life of 100 years. The concrete is required to have good working properties and mechanical properties, and the trial strength reaches 1.15 times of the design strength.

**Figure 2:**
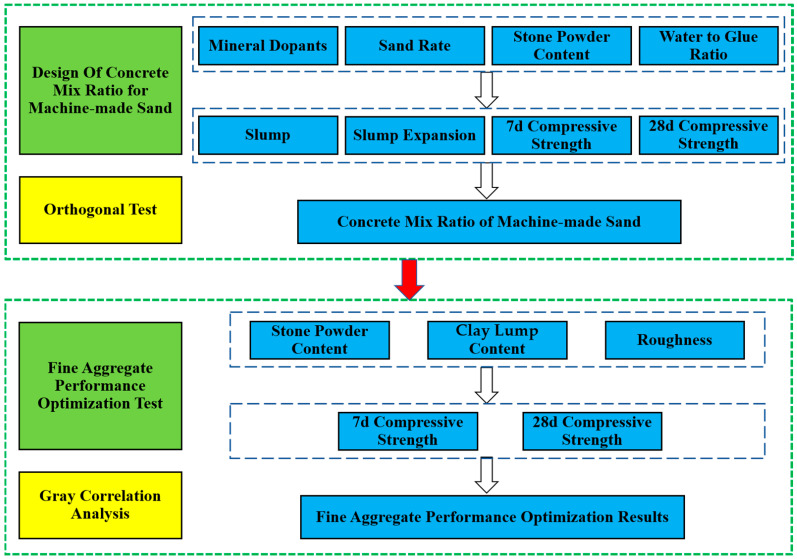
Flow chart of this study.

**Figure 3:**
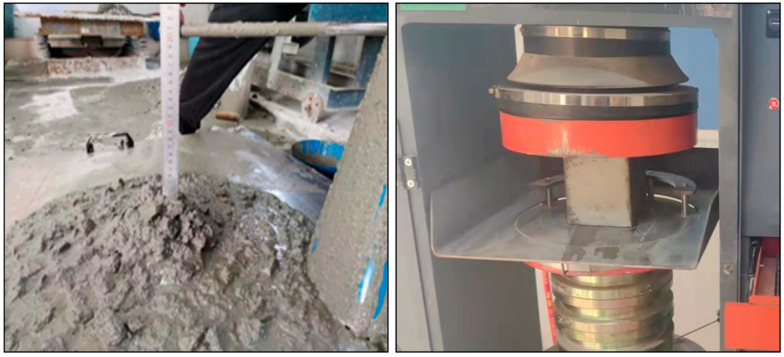
Field test chart of this study.

The working performance test indexes are slump and slump expansion. Additionally, the specific operation is carried out in accordance with the Chinese standard GB/T 50080-2016 “Standard for Test Methods of Properties of Ordinary Concrete Mixes”. The mechanical performance test indexes are compressive strength at 7 and 28 days. Additionally, the specimen size is 150 mm × 150 mm × 150 mm concrete test block, the test method is conducted according to the Chinese standard GB/T 50081-2002 “Standard on Mechanical Properties of General Concrete”. The process of this study is shown in Figure 2. The field test process is shown in Figure 3.

## 3. Results and Discussion

### 3.1. Test Results

Sixteen sets of concrete test blocks designed for orthogonal experiments were tested for working properties and mechanical properties. The results of the concrete slump and compressive strength tests are shown in Table 6.

### 3.2. Analysis of Extreme Differences

The orthogonal experimental design software Minitab 20 (State College, PA, USA) was used to perform a polar difference analysis on the experimental results. The extreme difference refers to the difference between the maximum value and the minimum value of the mean value of the test index corresponding to each level in each column. The effect of each level on the test indexes was ranked from largest to smallest by the extreme difference analysis. The results of slump and slump expansion analysis of concrete are shown in Table 7.

From Table 7, the influence of each factor on slump and slump expansion is ranked as sand rate > mineral admixture > stone powder content > water–cement ratio. With the increase in sand rate, the slump of fresh concrete tends to increase. There is an optimum ratio of coarse and fine aggregates for fresh concrete. In a certain amount of admixture, as the amount of mineral admixture increases, the concrete slump increases. This is due to the lubricating effect of fine particles of admixtures, so that the fluidity of fresh concrete increases. When the amount of admixture is too large, the amount of water needed to wrap the surface increases, resulting in a decrease in the fluidity of fresh concrete and a decrease in slump. With the increase in stone powder content, the concrete resistance to segregation is enhanced. The specific surface area of stone powder particles is large, and the water requirement for wrapping the powder surface is large. This leads to a decrease in concrete fluidity, but the cohesiveness and water retention of fresh concrete is good. With the increase in the water–cement ratio, the water consumption gradually increases, which to some extent promotes the fluidity and cohesiveness of fresh concrete.

The results of the 7-day and 28-day compressive strength extreme difference analysis of the machine-made sand concrete are shown in Table 8.

From Table 8, the degree of influence of each factor on 7-day compressive strength is as follows: mineral admixture > water–cement ratio > sand rate > stone powder content. The degree of influence of each factor on 28-day compressive strength is as follows: water cement ratio > sand rate > stone powder content > mineral admixture. The activity of fly ash in the mineral admixture is very low in the early stage, which does not participate in the reaction and has a weakening effect on the strength of concrete. With the growth of the age of concrete, the mineral admixture activity gradually emerges. However, some studies [38,39,40,41] pointed out that adding the right amount of mineral admixture can play its filling effect. It reduces the internal voids of concrete and increases the strength of concrete. When the mineral admixture is 20%, the strength of concrete has been significantly enhanced, and further increase the admixture has less effect on the improvement of concrete strength. With the increase in the water–cement ratio, the compressive strengths of concrete showed a gradual decrease. The increase in water consumption of concrete leads to an increase in the probability of excess water forming voids inside the concrete, thus gradually increasing the possibility of reducing the strength of concrete. To ensure the excellent mechanical properties of the machine-made sand concrete, a lower water–cement ratio should be used. The increase in fine aggregate content makes the cement paste more densely filled in the coarse and fine aggregates, which has the effect of enhancing the strength. However, when the sand rate exceeds a certain range, more slurry is required for the bonding surface of the concrete internal aggregate interface with the slurry, making the void rate between fine aggregates not completely filled by the slurry. The particle size of stone powder is between the cementitious material and the machine-made sand. Additionally, it can effectively fill in between the aggregate particles, forming a tightly packed body. However, when the stone powder content exceeds a certain range, it will reduce the fluidity of fresh concrete. Due to its lower activity, it will reduce the strength of concrete.

### 3.3. Single Factor Analysis

**Figure 4:**
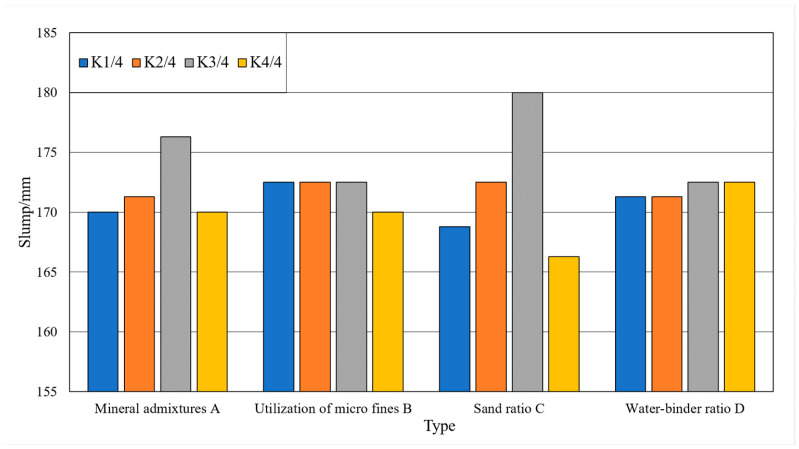
Trend of the influence of factors on slump test results.

**Figure 5:**
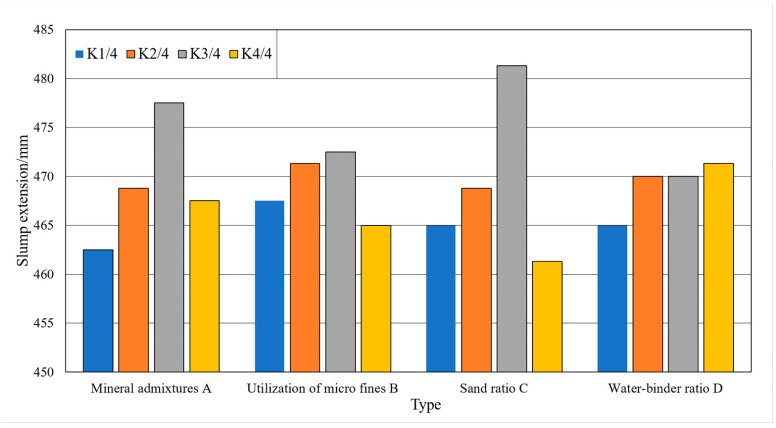
Trend of the influence of factors on the results of slump expansion test.

**Figure 6:**
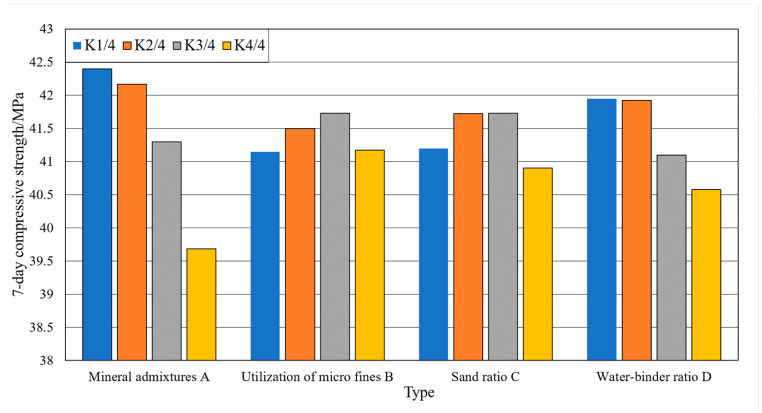
Trend of the influence of factors on the results of 7-day compressive strength test.

**Figure 7:**
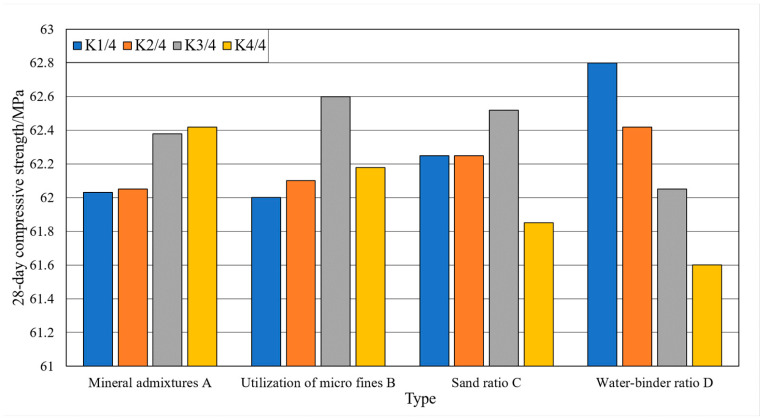
Trend of the influence of factors on the results of 28-day compressive strength test.

Single factor analysis refers to the situation where the test results vary with a single factor when other factors are constant. The analysis of the effect of single factor on the slump and slump expansion of the machine-made sand concrete is shown in Figure 4 and Figure 5. The analysis of the effect of a single factor on the compressive strengths of the machine-made sand concrete is shown in Figure 6 and Figure 7.

(1) The amount of mineral admixture

As shown in Figure 4 and Figure 5, with the gradual increase in mineral admixture, the concrete slump and slump expansion increase first and then decrease. The slump and slump expansion have great values at three levels. As shown in Figure 6 and Figure 7, the 7-day compressive strength of concrete gradually decreases with the increase in mineral admixture. The 28-day compressive strength is the opposite, gradually increasing with the increase in mineral admixture. However, the changes in 7-day compressive strength in the four levels are significantly greater than that of 28-day compressive strength. The growth of 28-day compressive strength is not obvious when the mineral admixture is mixed from 20% to 30%.

(2) Stone powder content

As shown in Figure 4 and Figure 5, the slump of the concrete keeps constant and then decreases with the increase in stone powder content. The slump expansion of concrete shows a trend of increasing and then decreasing. Due to the small particle size of stone powder, it acts as a lubricant in the fresh concrete system. Therefore, when the content of stone powder in the fine aggregate does not exceed a certain range, the fluidity and water retention of concrete has a certain role in promoting. As shown in Figure 6 and Figure 7, the compressive strengths show a trend of increasing and then decreasing with increasing stone powder content, with a great value at the third level. After the stone powder content exceeds 10%, the strength value decreases. However, the magnitude of the decrease is small.

(3) Sand rate

As shown in Figure 4 and Figure 5, the effect of sand rate on slump and slump expansion is similar to that of mineral admixture. With the increase in sand rate, it shows a trend of increasing and then decreasing. There are great values at all three levels. Additionally, the average value of different levels differs greatly. The maximum difference of slump and slump expansion are 13.8 mm and 20.0 mm. As shown in Figure 6 and Figure 7, the compressive strengths of concrete reached great values at all three levels. The difference of maximum difference of compressive strength between levels is small.

(4) Water–cement ratio

As shown in Figure 4 and Figure 5, the slump and slump expansion of concrete show an increasing trend as the water–cement ratio increases. Both of them have maximum values at level 4. As shown in Figure 6 and Figure 7, the compressive strengths of concrete change in the opposite pattern to slump and slump expansion. As the water–cement ratio increases, the compressive strengths show a decreasing trend. Unlike factors such as stone powder content, the increase in the water–cement ratio leads to a larger decrease in the 28-day compressive strength of concrete.

In summary, the optimal working performance of the machine-made sand concrete is achieved at a mineral admixture of 20%. Although the corresponding compressive strengths of concrete are not the respective maximum values, they differ from the maximum values by a small margin. The concrete slump and slump expansion are at maximum, and the compressive strengths of concrete are maximum when the sand percentage is 46%. When the stone powder content is 10%, the working performance is better, and it is in the state of imminent deterioration of performance. At this time, the compressive strengths have great values. With the increase in the water–cement ratio, the concrete slump and slump expansion show a gradual increase in trend, which has a certain improvement effect on the work performance. The water–cement ratio has a significant effect on the compressive strengths of concrete, and the strength decreases significantly as the water–cement ratio increases. Therefore, in order to ensure that the concrete has excellent mechanical properties, a lower water–cement ratio should be used as much as possible.

### 3.4. Experimental Validation

From the above points, it can be seen that the mineral admixture, sand rate and stone powder content are all at level 3 and the water–cement ratio is at level 1, i.e., A_3_B_3_C_3_D_1_. This is the optimal combination ratio of the machine-made sand concrete obtained from the orthogonal test. However, this group of tests was not available in the orthogonal tests. To further verify the accuracy of the results, three sets of tests were set A_3_B_3_C_3_D_1_, A_4_B_3_C_3_D_1_ and A_3_B_3_C_3_D_2_. The numbers are Y-1, Y-2 and Y-3. The working properties and mechanical properties of the machine-made sand concrete were tested. The matching ratio and test results of the verification experiments are shown in Table 9.

From Table 9, it can be seen that for the working performance, all three groups have better compatibility and are free from water secretion. The slump of both groups Y-2 and Y-3 is greater than that of group Y-1. Compared with the remaining two groups, the concrete of Y-1 group was slightly less fluid. The compressive strength test results showed that the 7-day compressive strength of concrete in Y-2 group was significantly smaller than that in the other two groups. 28-day compressive strength in Y-2 group was between the two. Compressive strengths of concrete in Y-1 group were the largest. 28-day compressive strength in Y-3 group was the smallest, but still reached the design strength requirement. In summary, the overall performance of the concrete of the three groups of the concrete with the machine-made sand is good.

### 3.5. Fine Aggregate Performance Test Results

**Figure 8:**
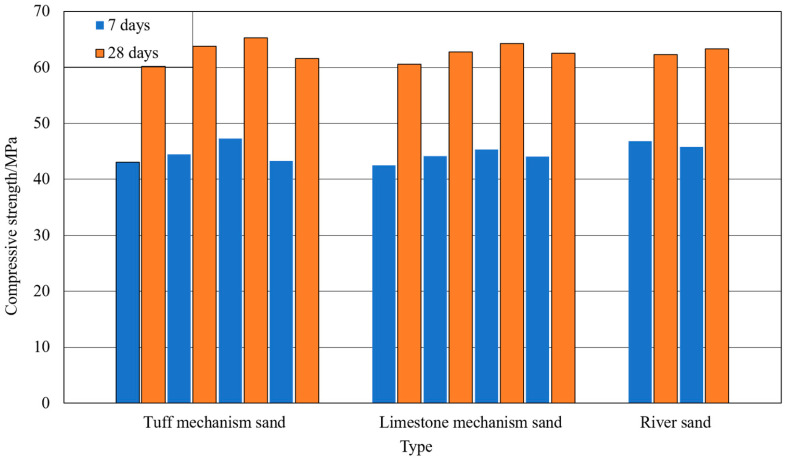
Compressive strength of concrete with different fine aggregate machine-made sand.

From Table 5, it can be obtained that the difference in bulk density and apparent density between the same fine aggregates is small. The loose bulk density and apparent density of machine-made tuff sand are close to that of river sand, but smaller than that of machine-made limestone sand. The fineness modulus of all batches of fine aggregates ranged from 2.6 to 3.0, which was in the range of medium sands. Roughness is a measure of the fluidity and angularity of fine aggregates. The experimental results showed that the roughness index values of the same fine aggregates differed less. Compared with the other two fine aggregates, the machine-made tuff sand has a rougher grain shape. Concrete was prepared using multiple batches of different types of machine-made sand and river sand based on the optimal combination of ratios. The performance of the concrete with different fine aggregates was compared. The specific mix parameters and test results are shown in Table 10. The variation of compressive strength of concrete with different fine aggregates is shown in Figure 8. (1_NH1_ denotes the first set of experiments, using fine aggregate of batch number NH1, and so on.)

Combining Table 10 and Figure 8, it can be obtained that the 28-day compressive strength of concrete of both tuff and machine-made limestone sand is greater than 60 MPa. The 7-day and 28-day compressive strength of concrete of both types of machine-made sand increases and then decreases with the increase in stone powder content and clay lump content. The compressive strengths of concrete were maximum for the 10% stone powder content of the machine-made sand. The compressive strengths of machine-made tuff sand fluctuated more. For the magnitude of concrete compressive strength variation, machine-made tuff sand concrete was significantly larger than machine-made limestone sand concrete. The maximum strengths of river sand concrete were slightly lower than those of the mechanism sand concrete, but still had good mechanical properties.

### 3.6. Grey Correlation Analysis

Grey correlation analysis is based on the similarity of the geometry of the series curves to determine whether they are closely related or not [42,43,44]. Grey correlation analysis is often used in concrete performance research. In exploring the analysis of the effects of stone powder content, clay lump content, roughness and mechanical properties of concrete of machine-made sand, the three subfactors are stone powder content, clay lump content and roughness. Two parent factors are 7-day and 28-day compressive strength of concrete, respectively. Each parent factor has three correlations to the three subfactors. The specific calculation steps are as follows.

1. Data column representation.

Specify the reference data column, which is often noted as $x_{0}$, i.e., the parent factor. The first case is denoted as $x_{0}\left(1\right)$, and the *k*th case is denoted as $x_{0}\left(k\right)$. The reference parent series can be expressed as $x_{0}$ = ($x_{0}\left(1\right),x_{0}\left(2\right),$…$,x_{0}\left(k\right)$).

2. Initialization process.

Before calculating the correlation coefficient, the series needs to be primed. Each number in the series can be divided in turn by the maximum value in the series to obtain a dimensionless data series for easy comparison.

3. Calculation formula of correlation coefficient and correlation degree.

Referring to the parent sequence $x_{0}$, with *k* comparison subsequences $\;x_{1}$,$\;x_{2}$, …,$x_{k}$, and the correlation coefficients are calculated as follows.

(1) $${\xi \;}_{i}=\frac{\underset{i}{\min}\underset{k}{\min}\left|x_{0}\left(k\right)-x_{i}\left(k\right)\right|+\;\rho \underset{i}{\max}\underset{k}{\max}|x_{0}\left(k\right)-x_{i}\left(k\right)|}{\left|x_{0}\left(k\right)-x_{i}\left(k\right)\right|+\;\rho \underset{i}{\max}\underset{k}{\max}|x_{0}\left(k\right)-x_{i}\left(k\right)|},$$

where  ρ is the resolution, which is used to reduce the error and improve the significance of the difference between the correlation coefficients. The value of 0.5 is taken in the paper. $\underset{i}{\min}\underset{k}{\min}\left|x_{0}\left(k\right)-x_{i}\left(k\right)\right|$ is the absolute value of the second-degree minimum difference between the $x_{0}\left(k\right)$ series and the $x_{i}\left(k\right)$ series at point k. $\underset{i}{\max}\underset{k}{\max}\left|x_{0}\left(k\right)-x_{i}\left(k\right)\right|$ is the absolute value of the secondary maximum difference between the $x_{0}\left(k\right)$ series and the $x_{i}\left(k\right)$ series at point k.

The degree of correlation is calculated by the following equation.

(2) $$r_{i}=\frac{1}{N}\overset{N}{\underset{k=1}{\sum }}{\xi \;}_{i}\left(k\right),$$

4. Construction of the strengths analysis matrix.

The correlations of the *m* parent factors and the corresponding *n* child factors are ordered in a row to obtain the correlation matrix *R*. The correlation matrix allows the dominance analysis of the parent and child series.

(3) $$R=\left(\begin{array}{cccc} r_{11} & r_{12} & \cdots & r_{1n}\\ r_{21} & r_{22} & \cdots & r_{2n}\\ \cdots & \cdots & \ddots & \cdots \\ r_{m1} & r_{m2} & \cdots & r_{mn} \end{array}\right),$$

Based on the experimental results in Table 10, the values of the subseries factors and the parent series factors for tuff sand and limestone sand concrete were primed. The results are presented in Table 11. The calculations were performed according to the steps in the grey correlation analysis to obtain the machine-made tuff sand correlation matrix *R*_(1__)_ and the machine-made limestone sand correlation matrix *R*_(2__)_.

(4) $$R_{\left(1\right)}=\left(\begin{array}{ccc} 0.5803 & 0.6137 & 0.8963\\ 0.5731 & 0.6065 & 0.8845 \end{array}\right),$$

(5) $$R_{\left(2\right)}=\left(\begin{array}{ccc} 0.5738 & 0.6088 & 0.9033\\ 0.5736 & 0.6085 & 0.9023 \end{array}\right),$$

A combined comparison of the machine-made tuff sand concrete correlation matrix *R*_(1__)_ and the limestone sand concrete correlation matrix *R*_(2)_,$r_{1i3}\;>\;r_{1i2\;}>\;r_{1i1}$ and $r_{2i3\;}>\;r_{2i2}\;>\;r_{2i1}\;$(*i* = 1, 2) indicate that the correlation between compressive strength and roughness at different ages is the largest. Additionally, the stone powder content has the least correlation. Among the three factors, roughness is the main influencing factor on the strength of the concrete of the machine-made sand. The greater the roughness of the concrete aggregate, the stronger the bond between the aggregates and between the aggregates and the cement paste. Concrete is about less prone to damage when compressed, and thus exhibits higher compressive strength. $r_{11j}\;>\;\;r_{12j}\;$ and $r_{21j}\;>\;r_{22j}\;$ (*j* = 1, 2, 3) indicate that the correlation between the 7-day compressive strength of concrete and roughness, stone powder content, clay lump content is greater compared to the 28-day compressive strength. As the early concrete fly ash has not been fully hydrated, the strength is low, and the enhancing effect of mineral admixture has not been fully developed. Therefore, the early strength of the machine-made sand concrete is more influenced by the roughness and mud lump content. $r_{1i1},\;\;r_{2i1}<0.6$ (*I* = 1, 2) indicate that there is no significant correlation between compressive strengths of concrete and stone powder content. When the stone powder t content does not exceed a certain range (about 10%), the stone powder content has little effect on the compressive strength of concrete. For ordinary concrete, the aggregate and cement paste interface is the high incidence of force damage. Additionally, it is dominated by damage to the bonding surface. The effect of stone powder on the bonding effect of aggregate and cement paste is small. Therefore, the stone powder does not improve the compressive strength of concrete significantly.

The performance of concrete specimens was determined by reducing the stone powder content and clay lump content of the machine-made tuff sand and blending the improved quality fine aggregate, Number NH5. Additionally, we compare it with NH2 batch of machine-made sand under the optimal combination of matching ratio. The results were also compared with the test results of groups 2 and 3. The apparent and bulk densities of the sand in groups 11 and 12 were not significantly different, and the fineness modulus was 2.7. The fine aggregates in groups 11 and 2 were from the same batch, and the performance index values were not significantly different. Other properties of fine aggregates and experimental results are shown in Table 12. (12_NH2_ indicates the 12th group of experiments, using NH2 batch of fine aggregates, others as well.)

From the test results, it can be seen that the compressive strengths of the concrete tested in group 12 are greater than the corresponding values in group 11. The index value of fine aggregate roughness in group 12 is greater than that in group 11 and slightly less than that in group 3. In addition, the difference between the compressive strength values of concrete in group 12 and group 3 is small. The experimental results are consistent with the findings of the study.

### 3.7. Application of Research Results

According to the concept of sustainable development, new environmentally friendly construction materials can protect the environment and save construction costs [24,25]. Qin et al. [44] used coral reef and coral sand as coarse and fine aggregates to prepare concrete samples in the context of offshore engineering. Additionally, they studied the uniaxial compressive strength of coral reef sand concrete. Shi et al. [31] studied the effect of lime powder in machine-made sand on the performance of concrete, and aimed at different stone powder content on the performance of concrete. A large number of studies have also focused on the development of new construction materials. This study is based on the background of a highway construction in eastern China, where river sand resources are scarce. However, tuff resources are abundant and raw materials for sand production are widely available. The use of tunnel rejects as sand making base material solves the problem of sand shortage in local projects and protects the local ecological environment. The concrete proportioning of tuff-derived sand was optimized by orthogonal test method. The optimal combination of concrete proportions was obtained. It was also verified that the concrete in the T-beam part of the bridge needs to have excellent performance. Only fine aggregates in concrete were investigated in this study, and environmentally friendly cementitious materials were not involved. In addition, the application of machine-made tuff sand concrete is also influenced by the local raw materials for sand production.

## 4. Conclusions

With the scarcity of natural sand resources, replacing natural sand with machine-made sand has become an inevitable choice for the sustainable development of concrete industry. In this study, the concrete performance of machine-made tuff sand is studied by orthogonal test and grey correlation analysis. From the experimental and analytical results, the following conclusions can be drawn.

1. The results of orthogonal tests were analyzed to study the degree of influence of each level on the test index. Among them, the sand rate has the greatest degree of influence on slump and slump expansion. The influence of mineral admixture dosing on 7-day compressive strength was the greatest. The water–cement ratio has the greatest influence on the 28-day compressive strength.
2. The optimal mix combination was obtained from the orthogonal test results. The optimal working performance of the machine-made sand concrete was achieved at the mineral admixture of 20%, sand rate of 46%, stone powder content of 10% and a water–cement ratio of 0.30. The performance of the concrete under the optimal mix combination was also verified.
3. We compared different fine aggregate concretes of similar quality. It is found that the mechanical properties of machine-made tuff sand concrete are similar to those of machine-made limestone sand concrete and river sand concrete.
4. Grey correlation analysis was applied to compare the factors influencing the mechanical properties of tuff and machine-made limestone sands. The correlation between compressive strength of concrete and roughness was found to be the highest. Additionally, the correlation with the stone powder content is the smallest. This points the way to quality control of high-performance machine-made tuff sand concrete.

Equal amounts of fly ash and slag powder were mainly considered as mineral admixtures in this study. We can analyze the effect of their different amounts on the properties of machine-made sand concrete in future studies. For the research of high-performance machine-made sand concrete, other environmentally friendly cementitious materials can also be considered.

## Figures and Tables

**Table 1:** Fine aggregate index properties.

Type	Fineness Modulus	Apparent Density/(g/cm^3^)	Bulk Density/(g/cm^3^)	Bulk Void Ratio/%	Stone Powder Content/%	Methylene Blue Value/(g/kg)	Roughness/(s)
Machine-made Tuff Sand (NH)	2.8	2.65	1.53	42.3	7.5	1.36	16.5
Machine-made Limestone Sand (SH)	2.7	2.71	1.67	38.4	6.4	1.34	13.4
River Sand (HS)	2.6	2.61	1.53	41.4	1.2	1.12	9.60

**Table 2:** L_16_(4^4^) orthogonal experimental design table.

	Factor	Mineral Admixture/%	Stone Powder Content/%	Sand Rate/%	Water Cement Ratio
Level					
1		10	3.0.	44	0.30
2		15	6.5	45	0.31
3		20	10.0	46	0.32
4		30	13.0	47	0.33

**Table 3:** L_16_(4^4^) orthogonal experimental scheme.

	Factor	Mineral Admixture A/%	Stone Powder Content B/%	Sand Rate C/%	Water Cement Ratio D
Test Number					
1		10	3.0	44	0.30
2		10	6.5	45	0.31
3		10	10.0	46	0.32
4		10	13.0	47	0.33
5		15	3.0	45	0.32
6		15	6.5	44	0.33
7		15	10.0	47	0.30
8		15	13.0	46	0.31
9		20	3.0	46	0.33
10		20	6.5	47	0.32
11		20	10.0	44	0.31
12		20	13.0	45	0.30
13		30	3.0	47	0.32
14		30	6.5	46	0.31
15		30	10.0	45	0.33
16		30	13.0	44	0.32

**Table 4:** Amount of each raw material for the test of machine-made sand concrete (kg/m^3^).

Test Number	Cement	Mineral Admixture	Machine-Made Sand	Stone	Water	Additive/%
1	471	52	779	975	157	1.5
2	471	52	794	971	162	1.5
3	471	52	810	950	167	1.5
4	471	52	824	930	173	1.5
5	444	79	792	968	167	1.5
6	444	79	772	982	173	1.5
7	444	79	832	938	157	1.5
8	444	79	812	953	162	1.5
9	419	104	824	930	173	1.5
10	419	104	827	933	167	1.5
11	419	104	777	988	162	1.5
12	419	104	797	973	157	1.5
13	366	157	830	935	162	1.5
14	366	157	814	956	157	1.5
15	366	157	789	965	173	1.5
16	366	157	774	986	167	1.5

**Table 5:** Main performance indicators of fine aggregates.

Test Number	Apparent Density/(g·cm^−3^)	Bulk Density/(g·cm^−3^)	Fineness Modulus	Stone Powder Content/%	Clay Lump Content/%	Roughness/S
NH1	2.65	1.52	2.8	3.0	0.2	17.5
NH2	2.64	1.53	2.7	6.5	0.4	16.4
NH3	2.65	1.52	2.6	10.0	0.8	18.6
NH4	2.67	1.53	2.6	13.0	0.9	19.7
SH1	2.71	1.65	2.9	3.0	0.2	14.6
SH2	2.70	1.64	3.0	6.5	0.4	15.7
SH3	2.71	1.68	2.8	10.0	0.7	17.1
SH4	2.72	1.67	2.6	13.0	0.8	16.3
HS1	2.65	1.49	2.7	1.6	0.7	11.9
HS2	2.66	1.52	2.6	1.3	0.9	10.6

**Table 6:** Machine-made sand concrete test results.

Test Number	Slump/mm	Slump Expansion/mm	7-Day Compressive Strength/MPa	28-Day Compressive Strength/MPa
1	165	450	42.5	62.5
2	170	465	43.3	62.4
3	180	480	43.0	62.0
4	165	455	40.8	61.2
5	175	470	41.9	61.9
6	170	470	41.5	60.8
7	165	460	42.5	62.9
8	175	475	42.8	62.6
9	185	490	40.5	62.1
10	170	470	40.6	61.8
11	175	480	41.9	63.2
12	175	470	42.2	62.4
13	165	460	39.7	61.5
14	180	480	40.6	63.4
15	170	470	39.5	62.3
16	165	460	38.9	62.5

**Table 7:** Slump and slump expansion degree extreme difference analysis table (mm).

Type	Level	Mineral Admixture A/%	Stone Powder Content B/%	Sand Rate C/%	Water Cement Ratio D
Slump	K_1_/4	170.0	172.5	168.8	171.3
	K_2_/4	171.3	172.5	172.5	171.3
	K_3_/4	176.3	172.5	180.0	172.5
	K_4_/4	170.0	170.0	166.3	172.5
	Polar Difference D	6.3	2.5	13.8	1.3
	Ranking	2	3	1	4
Slump Expansion	K_1_/4	462.5	467.5	465.0	465.0
	K_2_/4	468.8	471.3	468.8	470.0
	K_3_/4	477.5	472.5	481.3	470.0
	K_4_/4	467.5	465.0	461.3	471.3
	Polar Difference D	15.0	7.5	20.0	6.3
	Ranking	2	3	1	4

**Table 8:** Table of extreme difference analysis of compressive strength (MPa).

Type	Level	Mineral Admixture A/%	Stone Powder Content B/%	Sand Rate C/%	Water Cement Ratio D
7-day Compressive Strength	K_1_/4	42.40	41.15	41.20	41.95
	K_2_/4	42.17	41.50	41.72	41.92
	K_3_/4	41.30	41.73	41.73	41.10
	K_4_/4	39.68	41.17	40.90	40.58
	Polar Difference D	2.73	0.57	0.82	1.38
	Ranking	1	4	3	2
28-day Compressive Strength	K_1_/4	62.03	62.00	62.25	62.80
	K_2_/4	62.05	62.10	62.25	62.42
	K_3_/4	62.38	62.60	62.52	62.05
	K_4_/4	62.42	62.18	61.85	61.60
	Polar Difference D	0.40	0.60	0.68	1.20
	Ranking	4	3	2	1

**Table 9:** Test verification ratio parameters and test results.

Test Number	Proportion/(kg·m^−3^)						Test Result		
	Cement	Mineral Admixture	Machine-Made Sand	Stone	Water	Additive	Slump/mm	7-Day Compressive Strength/MPa	28-Day Compressive Strength/MPa
Y-1	419	104	814	956	157	1.5%	180	42.8	63.4
Y-2	366	157	814	956	157	1.5%	190	39.5	62.8
Y-3	419	104	812	953	162	1.5%	190	41.2	61.9

**Table 10:** Test mix ratio and test results.

Test Number	Proportion/(kg·m^−3^)						Test Result		
	Cement	Mineral Admixture	Machine-Made Sand	Stone	Water	Additive	Slump/mm	7-Day Compressive Strength/MPa	28-Day Compressive Strength/MPa
1_NH1_	419	104	814	956	157	1.6	180	43.1	60.2
2_NH2_	419	104	814	956	157	1.6	180	44.5	63.8
3_NH3_	419	104	814	956	157	1.7	170	47.3	65.3
4_NH4_	419	104	814	956	157	1.7	165	43.3	61.6
5_SH1_	419	104	814	956	157	1.6	195	42.5	60.6
6_SH2_	419	104	814	956	157	1.6	190	44.2	62.8
7_SH3_	419	104	814	956	157	1.7	180	45.3	64.3
8_SH4_	419	104	814	956	157	1.7	170	44.1	62.5
9_HS1_	419	104	814	956	157	1.6	200	46.8	62.3
10_HS2_	419	104	814	956	157	1.7	180	45.8	63.3

**Table 11:** Initialization results.

Test Number	Stone Powder Content	Clay Lump Content	Roughness	7-Day Compressive Strength	28-Day Compressive Strength
1_NH1_	0.2308	0.2222	0.8883	0.9112	0.9053
2_NH2_	0.5000	0.4444	0.8325	0.9408	0.9549
3_NH3_	0.7692	0.8889	0.9442	1.0000	1.0000
4_NH4_	1.0000	1.0000	1.0000	0.9154	0.9263
5_SH1_	0.2308	0.2500	0.8538	0.9382	0.9425
6_SH2_	0.5000	0.5000	0.9181	0.9757	0.9767
7_SH3_	0.7692	0.8750	1.0000	1.0000	1.0000
8_SH4_	1.0000	1.0000	0.9532	0.9735	0.9720

**Table 12:** Performance indicators and experimental results of high-quality fine aggregates.

Test Number	Stone Powder Content/%	Clay Lump Content/%	Roughness/s	7-Day Compressive Strength/MPa	28-Day Compressive Strength/MPa
11_NH2_	6.5	0.4	15.5	43.1	62.3
12_NH5_	3.0	0.1	18.3	46.2	65.8
2_NH2_	6.5	0.4	16.4	44.5	63.8
3_NH3_	10.0	0.8	18.6	47.3	65.3

## Data Availability

The data that support the findings of this study are available upon request from the authors.
